# A distinct negative leader propagation mode

**DOI:** 10.1038/s41598-021-95433-5

**Published:** 2021-08-10

**Authors:** O. Scholten, B. M. Hare, J. Dwyer, N. Liu, C. Sterpka, I. Kolmašová, O. Santolík, R. Lán, L. Uhlíř, S. Buitink, A. Corstanje, H. Falcke, T. Huege, J. R. Hörandel, G. K. Krampah, P. Mitra, K. Mulrey, A. Nelles, H. Pandya, J. P. Rachen, T. N. G. Trinh, S. ter Veen, S. Thoudam, T. Winchen

**Affiliations:** 1 Kapteyn Astronomical Institute, University Groningen, Landleven 12, 9747 AD Groningen, The Netherlands; 2 KVI Center for Advanced Radiation Technology, University of Groningen, Groningen, The Netherlands; 3 Interuniversity Institute for High-Energy, Vrije Universiteit Brussel, Pleinlaan 2, 1050 Brussels, Belgium; 4 Department of Physics and Astronomy, University of New Hampshire, Durham, NH 03824 USA; 5 Department of Space Physics, Institute of Atmospheric Physics of the Czech Academy of Sciences, Prague, Czechia; 6 Faculty of Mathematics and Physics, Charles University, Prague, Czechia; 7 Department of Astrophysics/IMAPP, Radboud University Nijmegen, Nijmegen, The Netherlands; 8 Astrophysical Institute, Vrije Universiteit Brussel, Pleinlaan 2, 1050 Brussels, Belgium; 9 Nikhef, Science Park Amsterdam, Amsterdam, The Netherlands; 10 Netherlands Institute for Radio Astronomy (ASTRON), Dwingeloo, The Netherlands; 11 Max-Planck-Institut für Radioastronomie, Auf dem Hügel 69, 53121 Bonn, Germany; 12 Institut für Astroparticle Physics, Karlsruhe Institute of Technology (KIT), P.O. Box 3640, 76021 Karlsruhe, Germany; 13 Erlangen Center for Astroparticle Physics, Friedrich-Alexander-Univeristät Erlangen-Nürnberg, Erlangen, Germany; 14 DESY, Platanenallee 6, 15738 Zeuthen, Germany; 15 Department of Physics, School of Education, Can Tho University, Campus II, 3/2 Street, Ninh Kieu District, Can Tho City, Vietnam; 16 Department of Physics, Khalifa University, PO Box 127788, Abu Dhabi, United Arab Emirates

**Keywords:** Natural hazards, Atmospheric dynamics

## Abstract

The common phenomenon of lightning still harbors many secrets such as what are the conditions for lightning initiation and what is driving the discharge to propagate over several tens of kilometers through the atmosphere forming conducting ionized channels called leaders. Since lightning is an electric discharge phenomenon, there are positively and negatively charged leaders. In this work we report on measurements made with the LOFAR radio telescope, an instrument primarily build for radio-astronomy observations. It is observed that a negative leader rather suddenly changes, for a few milliseconds, into a mode where it radiates 100 times more VHF power than typical negative leaders after which it spawns a large number of more typical negative leaders. This mode occurs during the initial stage, soon after initiation, of all lightning flashes we have mapped (about 25). For some flashes this mode occurs also well after initiation and we show one case where it is triggered twice, some 100 ms apart. We postulate that this is indicative of a small (order of 5 km$^2$) high charge pocket. Lightning thus appears to be initiated exclusively in the vicinity of such a small but dense charge pocket.

## Introduction

It has been known since the work of^1,2^ that negative leaders come in two categories, slower (known as $\alpha$ negative leaders) and faster ($\beta$ negative leaders). Fast negative leaders can approach propagation speeds on the order of a few $10^6$ m/s that have similar speeds as recoil leaders (which propagate down existing ionized channels). From a very extensive study of the existing evidence it was concluded in^3^ that negative leaders follow a continuous spectrum of speeds and one should not separate them in two classes. More recently^4–6^ argue that $\beta$ stepped leaders that initiate return strokes are a class apart. These create strong Very High Frequency (VHF) emission^4^, are followed by Elves^5^, and have a strongly increased luminosity^6^. Reference^7^ found that the vertical upward propagation speed of initial leaders of IC flashes is related to the initiation altitude. Their speed might reach $10^6$ m/s when started below an altitude of 6 km. In this work we show that a negative leader can ‘switch’ from a normal propagation mode (more like $\alpha$-leaders) to a mode where they propagate faster and emit intense VHF (30–80 MHz) and broadband radiation (more like $\beta$-leaders). In this paper we will refer to a leader in this second mode as an Intensely Radiating Negative Leader (IRNL). The IRNL mode is not necessarily associated with return strokes since we have observed them at initiation as well as during the development of a lightning flash without an associated return stroke. The IRNL mode is present only for a limited time after which normal negative leader propagation resumes, albeit that the number of negative leaders has increased considerably. During this particular phase of leader propagation we observe the emission of a copious amount of VHF radiation as well as strong pulses in a broadband antenna, installed at a distance of less than 10 km from the LOFAR core to support lightning observations.

In the work of^8^ the IRNL mode was already observed, but it was thought that this propagation mode, called an initial leader in that work, was exclusive to the initial stage of lightning development. The fact that in^9^ (see^10,11^ for an overview of the extensive literature on initial leaders) the transition from the initial leader to a negative stepped leader has been observed with high-speed video and in electric field change data, supports the finding that initial leaders and IRNL’s are the same. This is corroborated further by the observation of a correlation between a certain type of broadband radio pulses (called Initial Breakdown, IB) and VHF pulse amplitudes in^12,13^. In this work we show that an IRNL mode is not confined to the initial stage of a lightning (as is expressed by using a different name) and rather is evidence of a small but dense charge pocket. This charge pocket enhances the local electric field and thus facilitates lightning initiation.

In Dutch thunderstorms (thunderstorms imaged by the Dutch stations of LOFAR, the Low-Frequency Array^14^) the charge layers are usually at much lower altitudes than the storms measured in some parts of the US^15^. The main negative charge is at altitudes of 4–5 km with a positive charge layer below^16,17^. From the flashes we have imaged we infer that a large majority (more than 90%) of flashes we have imaged were initiated just below the negative charge layer with a downward negative leader that propagates towards the lower-lying positive charge layers.

In recent years radio observations of lightning has gone through some major developments, starting with lightning mapping arrays^18,19^ on to VHF radio interferometers^20–22^ and culminating in the precision observations using the LOFAR radio telescope operating in the 30–80 MHz band^8,17^. Superior results can be obtained with LOFAR because of its large number of antennas (about 200 are used in the measurements reported in this work), its large baseline (about 100 km), its high timing stability (better than 1 ns over a few seconds), and the possibility to retrieve the time trace of each antenna. These VHF observations are, however, not sensitive to currents at length scales of order 100 m and greater. To ameliorate this, a broadband magnetic-loop antenna^12^ was installed in the vicinity of the LOFAR core. These combined observations between LOFAR and a broadband antenna have facilitated the identification of the IRNL mode.

We show and analyze in “Data analysis” section the data for the two flashes for which simultaneous recordings are available from the magnetic loop antenna and LOFAR. A concluding discussion is presented in “Discussion and conclusions” section. In “Methods” section we give a short overview of the instrumentation and the imaging procedure.

## Data analysis

**Figure 1:**
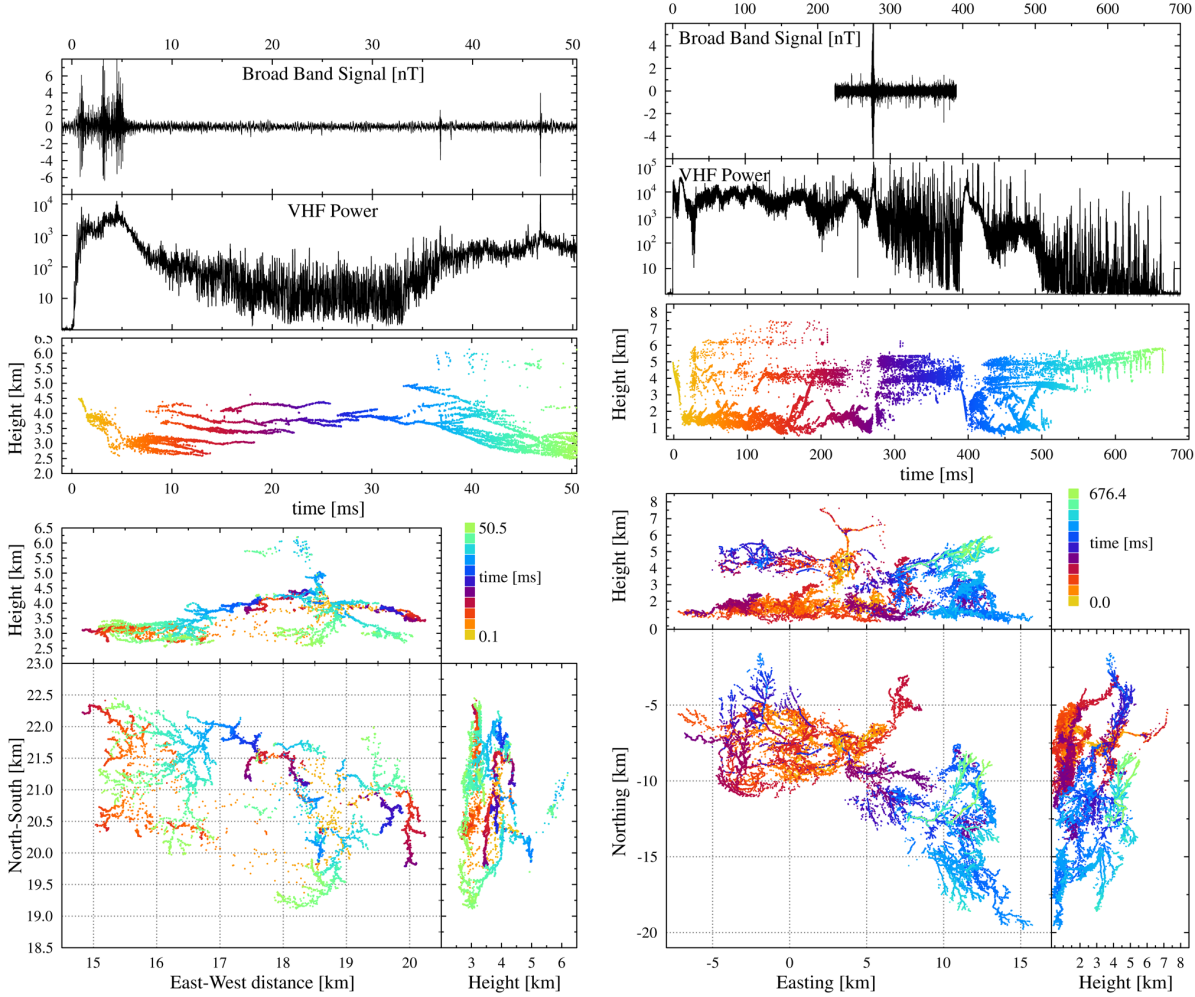
Left: Image of the first 50 ms of flash A showing sources with $\sigma (h)<3.5$ m, $RMS_d<5$ ns and $N_{ex}<55$. The topmost panel shows the recorded signal in the broadband antenna, re-binned over 0.2 $\upmu$s. The second gives the VHF power as measured by a LOFAR antenna in the core, re-binned over 20 $\upmu$s. The LOFAR noise power is given in units of the background-noise, see text. Right: Image of flash B showing sources with $\sigma (h)<3.5$ m, $RMS_d<3$ ns and $N_{ex}<55$.

**Figure 2:**
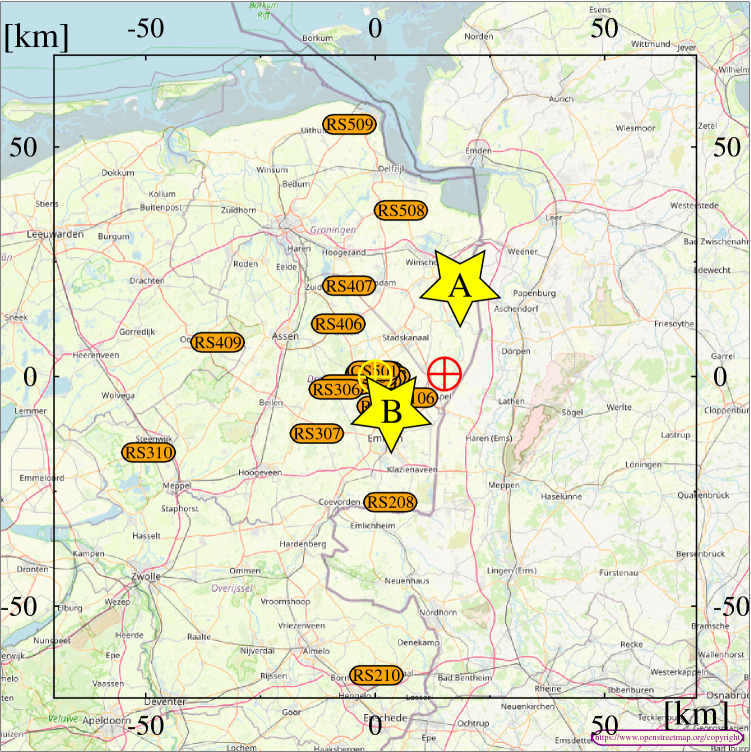
Layout of the Dutch LOFAR stations. The core of LOFAR is indicated by the yellow $\bigoplus$ sign, the magnetic loop antenna by the red $\bigodot$, while the yellow stars show the general location of flashes A and B that are discussed in this work. The map in the background is from OpenStreetMap^23^.

For two flashes we have recorded simultaneous measurements with LOFAR and the broadband antenna. Both flashes occurred on April 24, 2019 shortly after each other. The general structure of each of the two flashes can be seen from Fig. 1. Since in^8^ we have already shown the general structure of flash A, we focus here on the first 50 ms. The time calibration is such that $t=0$ is close to the beginning of the flash, 21:30:56.221 UTC for Flash A and 21:03:06.757 UTC for B. The general locations of the two flashes with respect to LOFAR are marked in Fig. 2.

From the structure of the leaders it can be inferred that for Flash A there was a positive charge layer below 4.5 km with a large number of negative leaders and a negative charge layer between 4.5–6.5 km height marked by the tell-tale twinkles^17^. For Flash B the negative charge layer lay much lower, below and around 2 km height. Twinkling positive leaders are seen in a band around 4 km height and also above 6 km from which it can be inferred that there were two negative charge layers.

The top two panels show the time traces as measured with the broadband antenna and the VHF power as measured by LOFAR. The broadband and LOFAR signal are aligned using the GPS timing of both systems. The VHF noise power is normalized to the background-noise, about $6\times 10^{-13}$ W/m$^{2}$, the strength of the signal in the absence of any lightning activity. This noise background is partly due to electronic noise in the LOFAR system and mostly due to galactic (extra terrestrial) radiation^24^. The data from the broadband antenna are available for only a part of each flash due to the fact that the broadband antenna stores traces of 167 ms length upon triggering.

**Figure 3:**
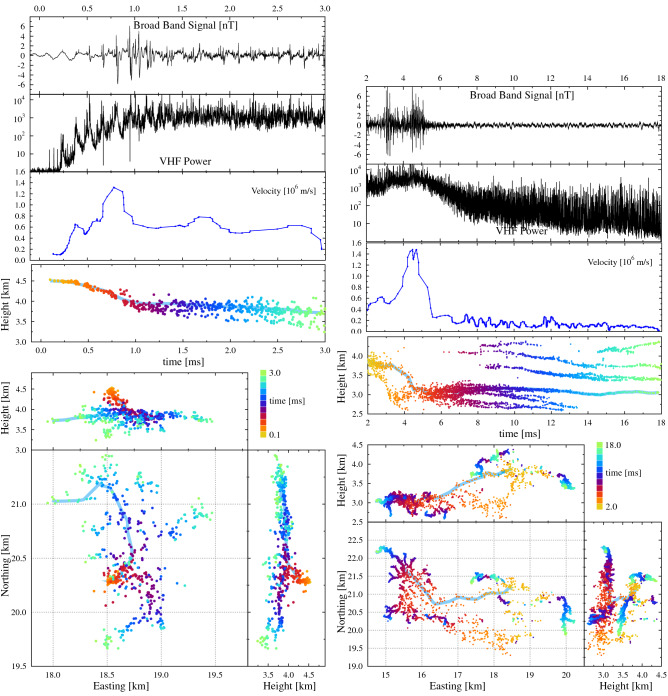
Similar to Fig. 1 with an added panel (third from the top) showing the propagation velocity along the track indicated in light blue in the lower panels. The power recorded at a core station of LOFAR is now re-binned over 2 $\upmu$s. The left (right) side pertains to the sources for the first 3 ms (2–18 ms) for Flash A, respectively.

In Fig. 3 the dynamics of the initial development of flash A is shown in detail. On the left the development in the very first 3 ms is shown, on the right the period from 2–18 ms. The panel structure of the figures is similar to that of Fig. 1, except that a third panel is added showing the propagation velocity of the leader along the track that is indicated in light-blue in the lower panels.

As shown in the lower panels on the left of Fig. 3, an initial leader propagated downward, immediately after initiation, from just below the negative charge layer at 4.5 km to the positive charge layer. The heights of these charge layers have been inferred from the structure of the observed leaders. As discussed in^8,25^ this initial leader has similar properties as those of a normal negative leader, however it propagates fast and quickly reaches a velocity of $10^6$ m/s, as can be seen from the velocity plot in Fig. 3. At the same time the VHF power increased by at least one order of magnitude and strong initial breakdown pulses were detected by the broadband antenna. Since we observe similar intense and fast negative leader at later times in the flashes, discussed further below, we refer to this remarkable mode in the development of a negative leader, lasting from about t = 0.7 to 1.7 ms, with the more appropriate generic name of “Intensely Radiating Negative Leader” (IRNL). When the positive charge region was reached at about t = 1.7 ms, the IRNL mode showed extensive branching and transitioned into a large number of more normal negative leaders (although still propagating at elevated velocities with elevated VHF emission). The total VHF intensity stayed large because it is the sum of the intensities of at least ten of these emerging negative leaders.

At shown on the right of Fig. 3, most of the negative leaders stalled at t = 3 ms (some of these got reactivated at later times) and only two continued propagating with elevated speeds (shown in the velocity panel for the upper of the two which is marked by the blue band). At t = 3 ms another surge in VHF power was detected as well as intense radio emission by the broadband antenna. The structures observed between t = 3 and 6 ms, intense radiation, fast propagation, diffuse distribution of sources with a multitude of emerging negative leaders, is a clear manifestation of a pair of leaders propagating in the IRNL mode. In Fig. 3 one of these is marked by the light-blue leader track (the track used to calculate the velocity follows the same leaders for both left and right sides where they are shown for different times). The velocity panel shows a clear peak of $1.5 \times 10^6$ m/s at the time the this negative leaders moved downward in the IRNL mode at t = 4 ms. The other negative leaders reached a similar speed at t = 3 ms in the IRNL mode.

When the negative leaders in the INRL mode propagated downward at high velocity, strong pulses were seen in the broadband antenna, consistent with the fact that this antenna is exclusively sensitive to vertical polarization as emitted by a vertical current. Although the density of broadband pulses is large, most of them are initially positive bi-polar pulses which is indicative of negative charge moving down (due to polarization of the antenna). This is a clear signature of a downward moving negative stepped leader with a large current moment. After fanning-out, the IRNL transitioned into a second batch of normal negative leaders at around t = 6 ms that then gradually slowed down to a typical propagation velocity of a negative leader of about $10^5$ m/s. In the velocity panel on the right of Fig. 3, the velocity of one such negative leader (as indicated by the blue band in the lower panels of Fig. 3) is given. A large number of negative leaders were all propagating simultaneously in an area of 1 $\times$ 2 km. After t = 6 ms, the time trace of the broadband antenna is rather feature-less while the average power in the VHF antenna gradually decreases by about 1 order of magnitude over the period t = 5.5–7.5 ms. At t = 7.5 ms we counted 25 distinct active negative leaders in a ring of about 1.5 km diameter centered at (N,E) = (21, 16) km. By t = 10 ms this number reduced to 15 in the same general area.

Based on the structures we observe in Fig. 3 the signatures of the IRNL mode are:

- It commences with the acceleration of a more normal negative leader or the acceleration of the negative leader formed at initiation.
- It reaches a speed of the order of $10^6$ m/s (much faster than normal negative leaders) in just a fraction of a millisecond.
- It is not propagating along an existing leader channel and thus is clearly distinct from a recoil or a dart leader.
- It is emitting strong pulses detected by the broadband antenna.
- It produces a strong increase in VHF power.
- There are fewer located VHF sources during this mode (as compared to typical negative leaders) by the imaging techniques used in this work.
- During the IRNL mode, propagation at top speed is observed for about 1–3 ms, covering distances of several km, after which it decelerates and creates a few dozen negative leaders that slow down to normal negative leader propagation speeds.
- In the image, broad filaments, or channels, can be distinguished with a width of the order of 100 m (much broader than normal negative leaders^26^) that tend to fan out to form a complex system spawning a large number of more normal negative leaders.

The IRNL mode is thus clearly distinct from normal negative leader propagation.

The small number of located sources during the IRNL mode is due to the fact that the time trace shows many partially overlapping VHF pulses that are difficult to locate with our imaging formalism. We are exploring interferometry-based (beam-forming) techniques to be able to image the sources of the many strong and partially overlapping VHF pulses. The stepping during the IRNL mode is seen most clearly at initiation (evident from the pulsed structure seen in the VHF power) when only one or two filaments (or channels) propagate in parallel. For the IRNL mode occurring later during the flash this stepping cannot be seen clearly. It may be due to the fact that there are too many propagating structures washing out the peak structure. It may also be that at the high velocity stage the propagation mode of these negative leaders becomes continuous rather than stepping.

**Figure 4:**
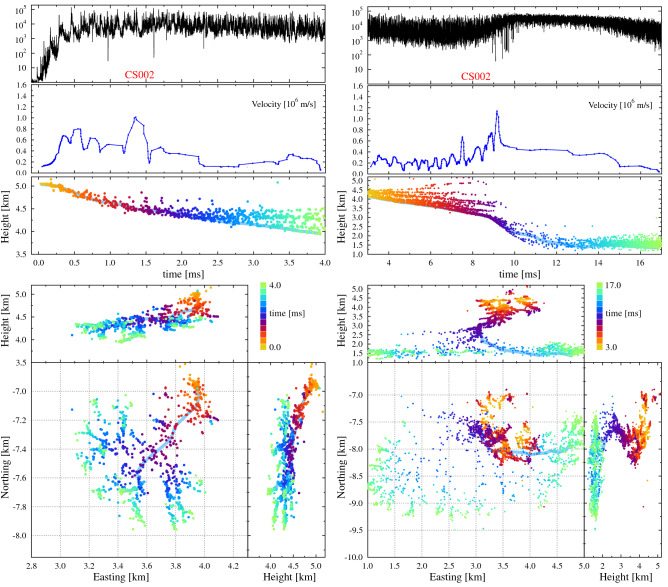
Same as Fig. 3 but for the initial parts of flash B, where the panels from top to bottom show the time trace, the velocity along a predefined track, and the altitude of the sources, all v.s. time. The bottom panels show different projections of the source positions. The predefined track is indicated by a light blue band. The left side of the figure shows the sources for the 4 ms of the flash, while the right for the subsequent 13 ms. The right side shows the sources on a more zoomed-out scale than the left.

Figure 4 shows the initial stage of Flash B, which also contained an IRNL, from t = 0.5 till 2.5 ms, indicating that the IRNL mode is common immediately after lightning initiation. Also this flash showed a second IRNL mode, in this case at 9–15 ms. Since the second IRNL occured a bit later in the flash, the velocity along the indicated track drops in between the two IRNL modes to values lower than seen in Fig. 3. Even though the velocity barely reaches $10^6$ m/s, and broadband data is not available, the features of the two IRNL modes are clearly recognizable, including: intense VHF emission, elevated speeds, low source density, broad channels that fan out, and copious number of produced negative leaders. The VHF power even saturates the LOFAR antennas, as seen between 10 and 16 ms in Fig. 4. The first IRNL for flash B spreads over an area of less than 1 km$^2$ while the second one covered an area of 2 × 3 km$^2$ at an altitude of only 1.5 km. The first IRNL, during the initial stage of the flash, show 6–7 high-power VHF pulses during the first millisecond, similar to what is seen in Flash A.

**Figure 5:**
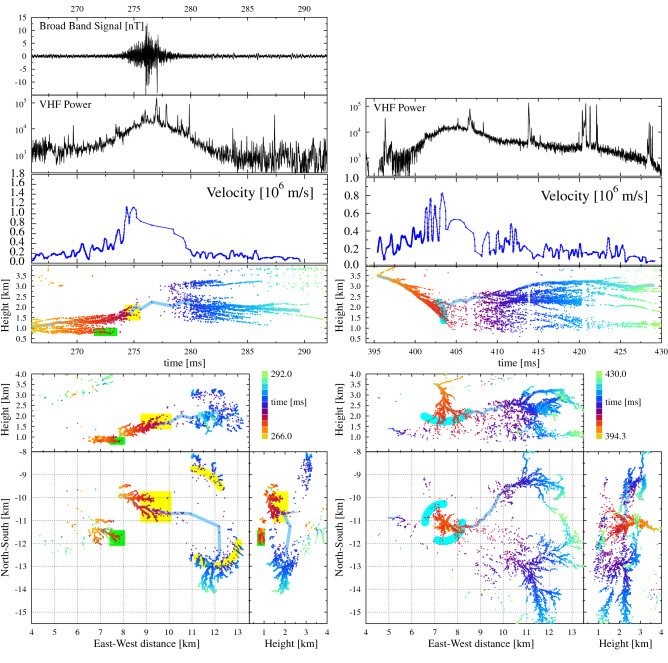
Same as Fig. 3 where the left shows a zoom-in for Flash B at the section where strong signals were measured in the broadband antenna, shown in the top panel, near t = 276 ms. The right side shows the time span of 395–430 ms of the same flash at the same location. For the later time there are no broadband data available. The scales for the left and right pictures are the same except for the time scale.

Most interesting is that the IRNL mode is not confined to the initiation region but may occur during the later development of the flash and appears to be linked to a particular spot in the atmosphere. The left-hand panel of Fig. 5 gives a zoom-in of Flash B around the time a strong signal was recorded by the broadband antenna and showing another IRNL from t = 274 till 280 ms. This figure shows two negative leaders that were propagating eastward, and then seemed to stop propagating, the first one, marked by the green box, at t = 272 ms at an altitude between 0.5 and 1.0 km and the second, marked by the yellow box, slightly later at t = 274 ms between (N = $-11$ to $-10$ km, E = 9–10 km) while propagating upward towards an altitude of 2 km. A bit later, after the strong pulses in the broadband antenna, around t = 279 ms the second negative leader seemed to re-appear in a broad semi-circle with a radius of about 3 km centered around the position where the second disappeared (indicated by the yellow arcs), to resume normal negative leader propagation. A few filaments can be distinguished in-between where the leader “disappeared” and resumed propagating. Based on the distance and time, the negative leader must have reached speeds of $10^6$ m/s when it was poorly imaged. During the same time span there is a strong increase in the emitted VHF power in the form of a very noisy, amorphous, structure with several short duration spikes superimposed on top. With the exception of the upper left, north-east, corner, no previous leader activity has been seen in the area depicted in Fig. 5. This is an archetypical example of an IRNL mode of propagation.

The right half of Fig. 5 shows the same area some 130 ms later during the same flash. For this period there are no data available from the broadband antenna. Figure 5 shows a negative leader propagating straight downward from the negative charge layer at 4 km into the positive charge layer at and below 2 km altitude. This occurred at the same location, projected on the ground plane, as where the first negative leader on the left side of the figure stalled. At t = 403 ms the structure of the imaged leader changes, as indicated by the cyan colors, to a structure reminiscent of that of an IRNL. 5 ms later, at a distance of about 4 km, normal negative leaders are imaged again, amounting to a speed of $8\times 10^5$ m/s. The northern branch (which is followed for making the velocity plot) propagates slightly slower. The VHF power shows a strong increase when the negative leader was propagating downward from 3.5 km height. There are no separate surges in VHF power visible to indicate stepping, which might be due to the fact that there were several IRNL branches active at the same time. Unfortunately there is no broadband time trace available for this time period so the stepping nature could not be confirmed. The lack of broadband data not withstanding, the picture shows another typical example of an IRNL.

As discussed above, the VHF power shows strong spikes on top of the broader emission from the IRNL in Fig. 5. At first glance, the source of these sharp spikes is unclear and they could be indicative of some IRNL physics. However, further investigation has shown that these spikes are actually due to positive leader activity very close to the LOFAR core (5 km height, 4 km horizontal distance), thus give rise to a very strong. Since the LOFAR antennas are bend-dipole antennas they have a large antenna gain for near-zenithal angles. Since these spikes are due to positive leader activity, and not the IRNL, we leave their investigation for possible future work.

Both sides of Fig. 5 strongly suggest that in the region of (N,E,h) = ($-13$ to $-9$, 5–12, 1–2.5) km there was a positive-charge pocket with a large density. This attracted the upper branch of the negative leader shown at the left in Fig. 5. A close inspection of the pulses seen in the broadband antenna at 274–276 ms shows that they have positive initial polarity corresponding to negative charge moving upward, consistent with the propagation direction during the IRNL mode as inferred from the located sources in Fig. 5. Note that the interpretation of the polarity is opposite to that of flash A since this flash is located at the opposite side of the broadband magnetic-loop antenna. We observe an accelerated propagation of especially the (yellow) branch at (N,E) = ($-10.5$, 9.5) km from $2\times 10^5$ m/s at t = 269 ms till $10\times 10^5$ m/s at t = 275 ms (see velocity panel). The propagation of this second branch apparently quenched the propagation of the first (green) branches, probably due to the large amount of negative charge present at its tip. At this point the charge must have been such that it could propagate at high speed during the IRNL mode with strong emission in the lower frequencies as picked-up by the broadband antenna. A bit later the negative leader decelerated again to $1.5\times 10^5$ m/s at t = 287 ms. This first leader complex appeared not to have neutralized the charge reservoir^27^ as about 100 ms later (right side of Fig. 5) another negative leader emerged from the negative charge layer at 3.5 km to enter the same local charge region. In this case the charge cloud was reached from above instead of from below. There seems to be a repeat of the processes seen earlier with a large number of negative leaders re-appearing at distances of 3–4 km. It is interesting to see that normal negative leaders are now visible at, for example, (N,E) = ($-11.5$, 12) km where in the earlier discharge only the IRNL mode was visible. Apparently this earlier discharge process had reduced the space charge to a sufficient extent to make normal negative leader propagation possible.

## Discussion and conclusions

In this work we have used the infrastructure of the LOFAR radio telescope to provide high-resolution 3D images of two flashes, supplemented with time traces of a broadband antenna. These combined observations allow us to recognize a negative leader propagation mode that occured multiple times in the two flashes. Since this mode is associated with a strong enhancement of VHF emission and the emission of strong pulses as detected by the broadband antenna, in this work we call it an “Intensely Radiating Negative Leader” mode. This mode has previously been referred to as an initial leader, but we have shown that it can occur later in the flash as well. We show that it lasts for a few milliseconds, shows elevated speeds, strongly elevated VHF and broadband emissions, and a ‘fuzzy’ image while the negative leaders, emerging in copious numbers, are well focussed.

During an IRNL we observe a more normal stepped negative leader accelerating quickly, within a millisecond, to speeds of order $10^6$ m/s while it fans out to cover (in plan view) an area of several square kilometers. When it spreads, often some wide channels are recognizable, although this is not always the case as sometimes the density of located sources is too small. The large VHF power is due to an almost continuous stream of overlapping strong pulses which is difficult to handle for our pulse-location algorithm. It will thus be interesting to apply an interferometry/beamforming based imaging procedure to obtain more insight in the propagation during the IRNL mode, which is planned for a future publication.

The IRNL mode occurs in relatively small volumes that are most likely the sites of small but dense pockets of charge. The IRNL mode thus is activated as the negative leader enters a region with a strong electric field due to this local dense charge cloud. The fact that at the end of the IRNL mode a large multitude of negative leaders have been created supports the picture of a small region with a very high space charge. The dense charge pocket is probably created by a local turbulence^28^, with a typical size of order 5 km$^2$. This dense space charge leads to a larger than usual amount of charge at its tip and an increased propagation velocity, both contributing to a strongly enhanced VHF power and strong broadband pulses when the propagation is in the vertical direction during the IRNL mode. Recently reference^29^ reported on a direct relation between conductivity and propagation speed which supports our interpretation. They show that an increased charge in the leader tip would imply larger currents and thus higher temperatures and free-electron densities. The enhanced charge may also be responsible for the very broad propagating structures that are imaged as ‘fuzzy’ channels in our procedure. It may even be that we have entered a regime where the charge is large enough for a non-stepping negative leader.

The finding that a mode of negative leader propagation hitherto known as initial leader is in fact not a feature tied to the initial development of a lightning flash but more general (therefore named IRNL in this work) and is instead likely due to the presence of a relatively rare small high-charge pocket, sheds important light on the mystery of lightning initiation^30^. Airplane and balloon observations^31,32^ have shown that the electric fields in thunderclouds is generally well below dielectric breakdown, much too low to ignite a lightning flash. However, it has been speculated^33,34^ that there could be local pockets of strong electric fields that are too local to easily measure. This work strongly suggests, for the first time, that thunderstorms indeed has spatially small high-strength regions of the electric field, in line with the findings of theoretical work such as^35^, among others, that particularly large electric fields are required for initiating lightning.

## Methods

The imaging procedure uses the time-traces recorded with LOFAR, see “LOFAR” section. We use the same imaging technique as described in^8^ where some of the essential steps are summarized in “Lightning imaging” section. The broadband magnetic loop antenna is described in some detail in “Broadband measurements with a magnetic loop antenna” section. The procedure used to calculate velocities is summarized in “Determining leader velocities” section.

### LOFAR

LOFAR^14^ is a radio telescope consisting of several thousands antennas primarily built for radio-astronomy observations. These antennas are spread over many European countries. The low-band antennas operate in the 30–80 MHz frequency band we use are bend-dipole antennas, installed in pairs oriented in the NE-SW and NW-SE directions. With this dual-polarized antenna pair the full polarization can be determined for an incoming plane wave. The Superterp is the core of LOFAR, it contains a dense array of antennas and is located near Exloo, the Netherlands. For radio-astronomy observations, the antenna signals are added coherently to effectively operate as a gigantic radio dish antenna with a diameter of 1000 km when the international stations are included.

From each LOFAR station we use 6 dual-polarized low-band antennas sensitive to 30–80 MHz. Each antenna samples at 200 MHz and has a 5 s long buffer. Using only the Dutch stations we reach baselines of up to 100 km (see Fig. 2). Upon an external trigger, the antenna buffers are frozen and data are read out. Sometimes some data is lost during during downloading, however this does not really affect the image quality due to the large number of available antennas (typically 300–400). For every 5 s of raw data from the TBBs we store close to 1 TB of data for later off-line processing.

### Lightning imaging

For completeness we outline here the impulsive imaging procedure we followed, see also^8^ where a much more detailed description given. The source code used for the analysis can be found at^36^. The procedure is a mixture of an interference and a time-of-arrival approach, where arrival time differences of a pulse at different antennas are obtained from the peak in the cross correlation between two antennas. The location of the source is obtained using a Levenberg-Marquardt fitting algorithm^37^ for the arrival-time differences.

Three quality indicators are used to ensure that the sources are well located. The most important is the obtained value for the Root Mean Square time difference between the calculated and measured arrival time differences ($RMS_d$) of the fit. Other indicators are the error on the height, $\sigma (h)^2$, which is usually the largest diagonal matrix element of the covariance matrix that corresponds to the error in determining the altitude of the source, and the number of excluded antennas, $N_{ex}$. An antenna is excluded from a fit for a particular source when the deviation is particularly large or when the shape of the cross correlation time trace deviates too much from the self correlation of the pulse in the reference antenna. This may happen when for a particular antenna the pulses emitted from two different sources arrive (almost) simultaneously.

### Broadband measurements with a magnetic loop antenna

A Shielded Loop Antenna with a Versatile Integrated Amplifier (SLAVIA)^12^ has been installed by the Department of Space Physics, Institute of Atmospheric Physics of the Czech Academy of Sciences at a site which is 0.6 km to the North and 15.1 km to the East with respect to the LOFAR core, close to the village of Ter Wisch, shown by a red $\bigodot$ in Fig. 2. The sensor measures the broadband waveform of the horizontal component of the time derivative of the magnetic field vector at a sampling frequency of 200 MHz. The maximum of its cosine shaped sensitivity pattern is in the north-northwest direction at an azimuth of 330$^\circ$. This means that an upward current pulse (moving negative charge downward) located at this azimuth from the loop will produce the largest positive pulse in the magnetic field waveforms obtained by numerical integration. The recording system has a sensitivity of 6 nT/s/${\sqrt{Hz}}$, corresponding to 1 fT/${\sqrt{Hz}}$ at 1 MHz. The data acquisition is not continuous: a snapshot with a duration of 167 ms is triggered when the absolute value of the derivative of the magnetic field exceeds a threshold of 23.3 mT/s. Every snapshot also includes a 52 ms long history before the trigger time. At the Ter Wisch site, the signal is unfortunately affected by strong man-made interferences and the waveform had to be cleaned by additional numerical narrow band rejection filters with bandwidths 18–30 Hz at interference frequencies between 2 and 10 kHz, and at 18 kHz (17 filters for the 21:03 event, 19 filters for the 21:30 event).

### Determining leader velocities

Extracting leader velocities is complicated by the fact that leaders branch. This makes it ambiguous to determine which sources should be grouped together to calculate the velocity along the main branch. The distance between sources is small enough that they frequently do not occur in time-sequence along the leader. This may be due to physics, i.e. VHF is not only emitted at the propagating leader tip but a few meters behind the propagating tip, or this may be due to source-location inaccuracies.

From the general structure of the leader first the leader track is drawn, as for example shown by the light blue band in the lower panels of Fig. 3. The velocity can now be calculated from the mean leader-tip location, determined by averaging the position of the sources with gaussian weighting factors depending on the source times i.e. sources that occur much later or earlier have small weights. For this work we have chosen a $\sigma =0.05$ ms for the averaging. Only sources within 50 m of the predefined leader track are included in the procedure. The more narrow peaks seen is the velocity panels of Fig. 3 could be an artifact due to side-branches off the main structure.
